# Cardiovascular Involvement in Pediatric *FLNC* Variants: A Case Series of Fourteen Patients

**DOI:** 10.3390/jcdd9100332

**Published:** 2022-09-30

**Authors:** Anwar Baban, Viola Alesi, Monia Magliozzi, Giovanni Parlapiano, Silvia Genovese, Marianna Cicenia, Sara Loddo, Valentina Lodato, Luca Di Chiara, Fabiana Fattori, Adele D’Amico, Paola Francalanci, Antonio Amodeo, Antonio Novelli, Fabrizio Drago

**Affiliations:** 1 Pediatric Cardiology and Arrhythmia/Syncope Complex Unit, Bambino Gesù Children’s Hospital, IRCCS, 00165 Rome, Italy; 2 Laboratory of Medical Genetics, Translational Cytogenomics Research Unit, Bambino Gesù Children’s Hospital, IRCCS, 00165 Rome, Italy; 3 Pediatric Cardiac Intensive Care Unit, Department of Cardiology and Cardiac Surgery, Bambino Gesù Children’s Hospital, IRCCS, 00165 Rome, Italy; 4 Unit of Muscular and Neurodegenerative Disorders, Bambino Gesù Children’s Hospital, IRCCS, 00165 Rome, Italy; 5 Department of Pathology, Bambino Gesù Children’s Hospital and Research Institute, IRCCS, 00165 Rome, Italy; 6 Heart Failure and Transplant, Mechanical Circulatory Support Complex Unit, Bambino Gesù Children’s Hospital, IRCCS, 00165 Rome, Italy

**Keywords:** *FLNC* and children, restrictive cardiomyopathy and major musculoskeletal changes, *FLNC* and congenital heart defects

## Abstract

Filamin C is a protein specifically expressed in myocytes and cardiomyocytes and is involved in several biological functions, including sarcomere contractile activity, signaling, cellular adhesion, and repair. *FLNC* variants are associated with different disorders ranging from striated muscle (myofibrillar distal or proximal) myopathy to cardiomyopathies (CMPs) (restrictive, hypertrophic, and dilated), or both. The outcome depends on functional consequences of the detected variants, which result either in *FLNC* haploinsufficiency or in an aberrant protein, the latter affecting sarcomere structure leading to protein aggregates. Cardiac manifestations of filaminopathies are most often described as adult onset CMPs and limited reports are available in children or on other cardiac spectrums (congenital heart defects—CHDs, or arrhythmias). Here we report on 13 variants in 14 children (2.8%) out of 500 pediatric patients with early-onset different cardiac features ranging from CMP to arrhythmias and CHDs. In one patient, we identified a deletion encompassing *FLNC* detected by microarray, which was overlooked by next generation sequencing. We established a potential genotype–phenotype correlation of the p.Ala1186Val variant in severe and early-onset restrictive cardiomyopathy (RCM) associated with a limb-girdle defect (two new patients in addition to the five reported in the literature). Moreover, in three patients (21%), we identified a relatively frequent finding of long QT syndrome (LQTS) associated with RCM (n = 2) and a hypertrabeculated left ventricle (n = 1). RCM and LQTS in children might represent a specific red flag for *FLNC* variants. Further studies are warranted in pediatric cohorts to delineate potential expanding phenotypes related to *FLNC*.

## 1. Introduction

Cardiomyopathies (CMPs) are a heterogeneous group of pathologies affecting the myocardium through electrical and/or mechanical dysfunction. Their severity and phenotypic manifestations are extremely variable, even within the same family members, due to high etiologic heterogeneity. Incomplete penetrance and variable expressivity are well described components that complicate phenotypic and genotypic characterization. The outcome is often unpredictable due to modulation by the environmental, epigenetic factors, genetic polymorphisms, and susceptibility loci. Based on the morphological and structural changes observed in the affected subjects, CMPs are classified as dilated (DCM), hypertrophic (HCM), restrictive (RCM), arrhythmogenic (ACM), and left ventricular non-compaction (LVNC) [1,2].

Despite their different morphological and pathological aspects, the underlying molecular basis can overlap, often involving the same biological pathways [3]. In this regard, cardiomyocyte hypertrophy is commonly observed in both DCM and HCM, whereas apoptosis can be observed in DCM and ACM [4,5]. Moreover, apoptosis can lead to fibrosis as a consequence of fibroblast activation and collagen deposition, also resulting in ACM forms [6].

Mutations in >170 genes are associated with different CMPs and channelopathies, both isolated and syndromic [7], but an increasing number of likely causative genes are reported, due to the extensive use of exome sequencing analysis in large cohorts of affected subjects.

The filamin C gene (*FLNC*) is located on the 7q32.1 chromosomal region and includes 48 exons [8]. *FLNC* presents two main transcripts (NM_001127487.2, NM_001458.4), which differ by the presence or absence of exon 31 [9,10]. The role of the two isoforms is not fully understood. The longest transcript (NM_001458.4) encodes a protein of 2.725 amino acids particularly expressed during cardiac stress. The shortest transcript (NM_001127487.2) is mainly present in normal situations and in skeletal muscle [11].

*FLNC* encodes for filamin C, a protein involved in myocyte function by interacting with both sarcolemma proteins and intercalated disks, reorganizing the actin cytoskeleton in response to signaling events and, probably, having structural functions at the Z-lines. It is involved in connections between cells and the extracellular matrix by binding to adhesion molecules, as well as in the organization of sarcomeres, presumably having a role in titin and actin anchorage to the Z-bands [7]. It consists of three functional domains: an N-terminal filamentous actin-binding domain (ABD), 24 immunoglobulin (Ig) domains divided into ROD1 and ROD2 sub-domains, and a C-terminal dimerization domain [12].

Functionally, filamin C necessitates the dimerization of two identical proteins through an Ig-like domain 24 interaction. Filamin C interacts with part of the Z-disc, signaling molecules, sarcolemma associated proteins, sarcoglycans, Xin actin-binding repeat-containing proteins (XIRP), and aciculin (mediated by a unique insertion in the Ig-like domain 20). A different organization is present between ROD1 and ROD2 domains, modulating ligand interaction [13,14,15,16,17,18].

Due to its complexity and involvement in many different biological processes, filamin C is reasonably associated with different phenotypes, depending on mutation type and localization, ranging from myopathies (myofibrillar distal or proximal myopathy) to isolated CMPs (RCM, HCM, DCM) [19]. In some cases, muscular and cardiac signs are both present in carrier subjects. *FLNC* variants are mostly reported in the adult population with little data available in pediatric cohorts. This paper aims to expand the *FLNC* spectrum, presenting previously described and novel variants associated with early-onset cardiac involvement, including CMPs, congenital heart defects (CHDs), and arrhythmias.

## 2. Materials and Methods

### 2.1. Clinical and Genetic Evaluation

A total of 500 unrelated pediatric patients with CMPs, arrhythmias, and CHDs were evaluated between 2015 and 2020 at our hospital and were tested by next generation sequencing (NGS) in silico panel of heart-related disease-causing genes, including *FLNC* (NM_001458.4). The probands and their parents provided written informed consent for genetic analyses.

### 2.2. Genetic Analysis

Genetic testing was performed at the genetic laboratories of the Bambino Gesù Children’s Hospital, IRCCS. DNA was extracted from peripheral blood with Qiagen columns (QIAamp DNA minikit; Qiagen, Hilden, Germany) according to the manufacturer’s instructions. The concentration and purity of the DNA samples were quantified by an ND-1000 spectrophotometer (NanoDrop; Thermo Scientific, Waltham, MA, USA) and FLx800 Fluorescence Reader (BioTek, Winooski, VT, USA).

### 2.3. Chromosomal Microarray Analysis

The chromosomal microarray analysis was performed using the Infinium CytoSNP-850K BeadChip (SNP-array, Illumina, San Diego, CA, USA) according to the manufacturer’s protocol. Array scanning data were generated on the Illumina iScan system, and the results were analyzed by Bluefuse Multi 4.4 software. Each detected copy number variant was evaluated considering its frequency on the healthy human population (DGV, Database of Genomic Variant), gene content, and scientific literature.

### 2.4. Next Generation Sequencing Analysis and Variant Interpretation

NGS analysis was performed on genomic DNA using the Twist Custom Panel (Clinical Exome Twist Bioscience) according to the manufacture’s protocol on the Illumina NexSeq550 or NovaSeq6000 platform (Illumina, San Diego, CA, USA). The reads were aligned to the human genome build GRCh37/UCSC hg19. The Dragen Enrichment application of BaseSpace (Illumina) and TGex software (LifeMap Sciences, Alameda, CA, USA) Geneyx Analysis (a knowledge-driven NGS analysis tool powered by the GeneCards Suite) were used for calling and annotating variants, respectively. Variants of the genes associated with CMPs (the list of analyzed genes is available in supplementary material data_S1) were scored and filtered by the TGex-Geneyx Analysis software. Among the evaluated variants by TGex-Geneyx and matching with the CMP phenotype, those meeting the following parameters were filtered: (1) nonsynonymous exonic or ±5bp intronic variants; (2) minor allele frequency (MAF) in the Genome Aggregation Database (GnomAD) of less than 0.01 (1%); (3) quality of the call variant: coverage ≥ 30X and GQ ≥ 50; and (4) at least 20% of reads showing the alternative allele (Alt > 20%). Variants were visualized by the Integrative Genome Viewer (IGV). Sequence data were carefully analyzed, and the presence of all suspected variants was checked in the public databases (gnomAD, dbSNP, 1000 Genomes Project, EVS, ExAC). An in silico prediction of variants’ pathogenicity was obtained using Sorting Intolerant from Tolerant (SIFT), Polymorphism Phenotyping v2 (PolyPhen-2), and Mutation Taster for the prediction of deleterious nonsynonymous single nucleotide variants for human diseases. The variants were evaluated by VarSome [20] and classified according to the American College of Medical Genetics and Genomics (ACMG) criteria [21].

The singleton’s variants were confirmed by Sanger sequencing following a standard protocol (BigDye Terminator v3.1 Cycle Sequencing Kit, Applied Biosystems by Life Technologies). Segregation analysis was performed when the relatives’ samples were available. The NGS workflow is available in Supplementary Material Data_S1, Figure S1.

### 2.5. Histology and Immunohistochemistry

Cardiac muscle tissue was obtained either from a right endomyocardial biopsy or the left ventricular (LV) myocardium at the time of LV assist device placement or heart transplantation (HT). Myocardial samples were processed according to standard histology protocols for hematoxylin and eosin and Masson’s trichrome.

Immunohistochemistry staining for *FLNC* was performed on formalin fixed and paraffin embedded myocardial sections. Immunoperoxidase staining for *FLNC* was performed using anti *FLNC* rabbit monoclonal antibody (Abcam EPR14498(B), Cambridge, MA, USA) (1:500) raised against the C-terminal peptide. Counterstaining for nuclei was performed with hematoxylin. Images were obtained with a Olympus BX53 microscope (Olympus, Tokyo, Japan).

Light microscopic analysis showed myocyte hypertrophy and a mild increase of interstitial fibrosis. The immunohistochemical staining of *FLNC* did not show a significant presence of aggregates in cardiomyocytes.

## 3. Results

**Figure 1:**
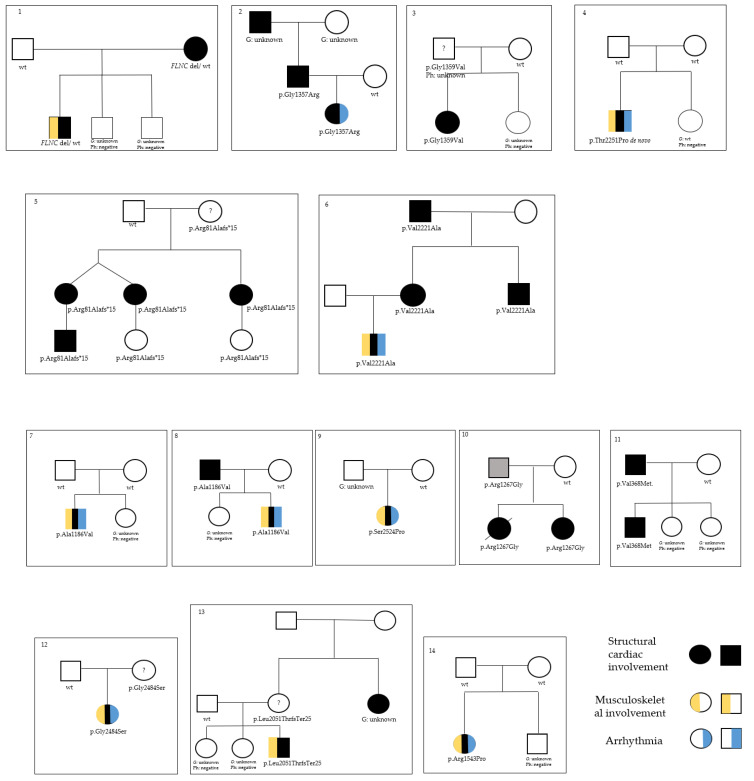
Family pedigrees of the study cohort (from patient 1 to patient 14). Please note that genotyping of relatives younger than 18 years with normal cardiac screening was not performed. Regular cardiac follow-up is offered but pre-symptomatic testing of healthy children is avoided.

Genetic analysis identified 13 rare variants and one copy number variant (CNV) in FLNC in 13 unrelated families (Figure 1): 12 variants in 13 patients detected by NGS analysis (10 missense and two truncating variants) and one CNV detected by SNP array analysis (Table 1). A trio analysis was performed on patients 5, 6, 7, 9, 10, 11, and 13, whereas a singleton NGS analysis was performed on patients 2, 3, 4, 8, and 12, with variants confirmed by Sanger sequencing (Supplementary Material Data_S1, Figure S2).

Among missense variants, only p.Ala1186Val and p.Gly2484Ser have been described previously in the literature [8,22], whereas the others are novel. According to ACMG guidelines, eight variants were classified as variants of uncertain significance (VUS) (class 3), four likely pathogenic (class 4), and one pathogenic (class 5).

The cardiac phenotype differed greatly in the nature and severity of manifestations (Table 1), including different forms of CMP, CHDs, and arrhythmic abnormalities. Moreover, the extracardiac phenotype was extremely variable, ranging from absent manifestations to obvious myopathic, skeletal, and connective tissue changes. The variants were de novo in three patients (no. 4, 7, and 14) and inherited from an affected relative in seven patients (no. 1, 2, 5, 6, 8, 10, 11). Father genotyping was missing in one patient (no. 9) and parent phenotyping was missing in three patients (no. 3, 12, 13), but patients 3 and 13 had a positive family history of CMPs.

The DCM spectrum included six patients: patient 1 had an arr[hg19] 7q32.1(128,470,838-128,521,431)x1 7q32.1 microdeletion; patient 2 (p.Gly1357Arg), patient 3 (p.Gly1359Val). Patient 4 (p.Thr2251Pro) showed increased left ventricular trabeculation and mild long QT syndrome (LQTS); patient 5 (p.Arg81AlafsTer15) had a positive family history of DCM and showed an ejection fraction at the lower limits of normal and diffuse late gadolinium enhancement (LGE) on cardiac magnetic resonance (CMR). Patient 6 (p.Val2221Ala) had a positive family history of DCM without structural abnormalities in the proband, except for a 2nd degree atrioventricular block (AVB).

Four patients showed early-onset severe RCM with major musculoskeletal involvement. Two of them carried the same variant: patients 7 and 8 (p.Ala1186Val) both on the HT waiting list. It arose de novo in patient 7, whereas in patient 8 it was inherited from a phenotypically positive father (musculoskeletal abnormalities and RCM). Patient 9, who underwent HT, carried the variant p.Ser2524Pro that was absent in the mother, and the father refused genetic testing. Patients 7 and 9 showed long QT but did not carry any pathogenic variants in major genes of LQTS. 

Two patients showed mild HCM [patient 10 (p.Arg1267Gly) and patient 11 (p.Val368Met)].

A wide spectrum of cardiovascular and arrhythmic involvement was observed in an interestingly heterogeneous group, with two patients having CHD: patient 12 (p.Gly2484Ser) with an atrial septal defect (ASD) associated with congenital complete AVB, and patient 13 (p.Leu2051ThrfsTer25) with a unicuspid aortic valve (UAV) and ascending aortic dilatation.

## 4. Discussion

CMPs are a heterogeneous group of diseases, which may lead to progressive heart failure or sudden cardiac death (SCD). They are rare conditions in the pediatric population: DCM is observed in 1:5000 children, HCM in 1:30,000, whereas RCM and ACM are considered extremely rare forms [24,25,26]. Familial cases are often autosomal dominant and are characterized by incomplete penetrance and variable expressivity.

*FLNC* is a well-studied gene responsible for multiple biological functions, firstly reported in association with a muscular phenotype and lately described in several types of adult onset CMPs. In a literature review, Kiselev et al. showed that different outcomes do not depend on the affected protein domain [22]. Mutation type is probably the most critical aspect to be considered when evaluating a genotype–phenotype association [8,22,27].

Three main mechanisms of action in the etiology of conditions related to *FLNC* variants may be observed: haploinsufficiency caused by a premature stop codon and nonsense-mediated decay, toxic gain of function that alter ligand binding properties, and a saturation of proteasome and autophagy pathways caused by misfolded proteins [8,15].

Several missense and in-frame indel mutations in *FLNC* [8,22,28], disrupting the normal organization of myofibrils and often leading to abnormal cytoplasmic filamin C aggregates, were described in a particular form of myofibrillar myopathy, consisting in late onset skeletal myopathy, with or without poorly characterized CMP. Missense variants are also described in HCM or RCM, without documented muscular involvement. Protein aggregates are usually observed in in vivo models overexpressing the aberrant protein and in most cases of a patients’ cardiac biopsy, depending on the patients’ age. In fact, proteins aggregates are not considered to be the cause of the clinical phenotype, which arises independently, but rather the reflection of the efficiency of the intracellular protein degradation system. Regarding non-truncating *FLNC* variants, knockout Zebrafish shows peculiar histological cardiac features in terms of protein aggregates in the perinuclear regions, irregular Z-discs, myofibrillar disorders, and reduced continuous sarcomeres. The subsequent separation of filamin C from the Z-discs results in myofibril disintegration. In addition, other studies in Zebrafish expressing filamin C show that the protein aggregates formed can recruit the molecular chaperone BAG3 and block other autophagy pathways. These studies suggest that BAG3 and the autophagy mechanism are potential targets of myofibrillar myopathy [29,30,31,32,33]. In contrast, haploinsufficiency-associated variants (inactivating/stop/frameshift variants) often hesitate in isolated CMPs. This may be due to a protein amount reduction affecting mechanical force transduction in the left ventricle, which is normally exposed to high mechanical force generation [23]. Recently, one of the largest multicenter studies in 85 patients with truncating *FLNC* variants (*FLNCtv)* has shown that this disorder is phenotypically heterogeneous, ranging from typical DCM to ACM. Left ventricular ejection fraction (LVEF) is related with the risk of organ endpoint and nonarrhythmic endpoints but not with the arrhythmic risk of SCD/major ventricular arrhythmias, which seems out of proportion in relation to the LVEF degree. In this respect, the 2019 Heart Rhythm Society consensus statement on ACM recommends the primary prevention of SCD with an indication for implantable cardioverter defibrillation therapy exclusively on the basis of LVEF < 45% (Class IIa recommendation) [34]. In the paper of Gigli and colleagues, SCD primary prevention is recommended independently of LVEF due to unpredictable arrhythmic events that may occur even with preserved LVEF. The authors recommended a personalized approach, emphasizing the need for larger studies to provide a tailored management [35].

Regarding CMP phenotype, the prevalence of *FLNC* variants in DCM was reported by Verdonschot et al. to be about 1–4.5% (mainly truncating and fewer missense variants) [8]. *FLNC* missense variants were also associated with HCM with a prevalence of 1.3–8.7% [8]. *FLNC* variants in RCM [7,22], ACM [24], and LVNC [12,36] have been reported less frequently.

In recent years, an expanding phenotype has progressively been emerging in *FLNC* variants in association with arrhythmias without structural alterations [37].

In our cohort, 12 *FLNC* variants and one *FLNC* CNV in 13 unrelated patients were identified: one microdeletion involving almost the whole coding sequence of *FLNC* (exons 2-48/48), nine missense variants, and two truncating variants. 

### 4.1. Microdeletion

The genomic 7q32.1 microdeletion was detected in patient 1, most probably determining a haploinsufficieny effect similar to truncating loss-of-function variants. The patient presented a severe form of DCM and LVNC. He inherited the 7q32.1 microdeletion from his mother who showed DCM as well. An NGS analysis was negative for significant variants. To the best of our knowledge, this is the first report of an *FLNC* genic deletion diagnosed by microarray. This finding highlights the importance of considering *FLNC* deletions in DCM cases tested negative at NGS; further analyses may be suggested, including multiplex ligation-dependent probe amplification, microarray, or specific NGS algorithms able to detect CNVs.

### 4.2. Missense Variants

The same p.Ala1186Val variant was detected in two unrelated patients (no. 7 and 8), presenting with RCM and limb-girdle multisystemic involvement with proximal weakness. This variant was previously described by Kiselev et al. in three patients showing a similar phenotype [22]. Two further reports are present in the scientific literature, even if the reported phenotypes are only focused on cardiac or muscular aspects, respectively. Xiao et al. [38] reported on one patient presenting with severe RCM but missing a musculoskeletal description, while Ghaoui et al. described a patient with limb-girdle dystrophy, but missing the cardiac phenotype [39]. This supports the importance of a multidisciplinary approach and clinical description of young patients for a proper genotype–phenotype correlation, which might be overlooked in case of “unispecialistic” evaluation.

The variant detected in patient 12 (p.Gly2484Ser) was recently reported by Verdonschot et al. in association with DCM and classified as VUS, but the age at DCM onset in this patient was not reported [8]. Our patient showed complete AVB and CHD (ASD). The latter defect has been previously described in a few patients harboring *FLNC* variants and should be considered among the *FLNC*-related phenotypes [22]. In previous case series [40,41], a consistent functional overlapping between CHD and CMP genes has been described. Emerging NGS data show how genes notoriously related to classical forms of CMPs can be associated with CHDs, expanding the knowledge of genotype–phenotype correlation [42]. No CMP signs have been observed in our patient (6 years of age). Her mother, a heterozygous carrier for the same variant, refused to perform the scheduled echocardiographic follow-up.

The remaining eight missense variants are not reported so far in the scientific literature and, based on ACMG guidelines, seven were classified as VUS with a possible damaging effect on protein, and one as a likely pathogenic variant.

-p.Gly1357Arg, detected in patient 2 and in her father, is annotated in the ClinVar database (ID: 654428). This patient showed end-stage heart failure secondary to DCM and mild musculoskeletal signs, whereas her father presented mild LV dysfunction and increased interventricular septum wall thickness.

-p.Gly1359Val was detected in patient 3, presenting with a very early onset (3 months of life) severe DCM and endomyocardial fibroelastosis with end-stage heart failure. She underwent HT and is currently on regular follow-up. The variant was inherited from the father, whose phenotype was unavailable.

-p.Thr2251Pro arose de novo in patient 4 who presented with LV hypertrabeculation, with a normal function and size, and mild long QT.

-p.Arg1267Gly, detected in patient 10 and her father, is annotated in the ClinVar database (ID: 930952). This patient showed a mild form of HCM. The variant was also detected on DNA extracted from the heart tissue of her sister, who died suddenly at 7 years old with an anatomic autopsy confirming HCM. The heterozygous carrier father did not show any signs of HCM, except for unexplained bradycardia.

-p.Val368Met was detected in patient 11 and in his father, both of them presenting with HCM. The variant is listed in the ClinVar database (ID: 471960).

-p.Val2221Ala was detected in patient 6 in addition to a variant in the *TTN* gene: c.13726G>T, p.(Glu4576Ter). Both variants were inherited from the affected mother. The patient presented only 2nd degree AVB without signs of structural myocardial involvement. The mother and grandfather, harboring the same variant, were diagnosed with DCM in the fifth and sixth decade of life, respectively. His maternal uncle, only harboring the *FLNC* variant, had acute heart failure at 49 years and presented hypertracebeculation and diffuse LGE. In this family, a double genic hit may be hypothesized, leading to CMP genesis, with a phenotypic modulation between *FLNC* and *TTN* variants. Notably, the maternal uncle carried only the *FLNC* missense variant, suggesting a specific role for *FLNC* in the pathogenesis of the disease in this family.

-p.Ser2524Pro was detected in patient 9 presenting with a severe RCM onset at 11 years and prolonged QT. She received HT. The variant was not maternally inherited. The unaffected father was not available for genetic analysis.

-p.Arg1543Pro was detected in patient 14 presenting with RCM onset at 12 years and a QTc at upper normal limits. She is on the HT waiting list. It is a de novo variant that has never been described in the literature but is classified as a likely pathogenic variant according to the ACMG guidelines.

Regarding the association of *FLNC* and LQTS, we identified a relatively high prevalence considering our small cohort (patients 4, 7, 9). LQTS is a heterogeneous condition. It can be associated with CHDs, electrolyte disturbances, and channelopathies. In spite of rapid advances in molecular diagnostics, detection rates remain as high as 60–70%. Genetically elusive patients might be due to unidentified molecular mechanisms, potential epigenetic factors, or other unknown mechanisms. There might be some explanations that include hemodynamic aspects in patients 7 and 9 who had RCM, including potential dilatation and “stress” over the conduction system. The association of *FLNC* with channelopathies has been described by Neethling et al. in 2016 who reported an interaction between *FLNC* and *KCNE2* (potassium voltage-gated channel) known to be causative for LQTS. In particular, they stressed this functional modulation under conditions of hypoxia and concluded that this pathway is likely to be involved in LQTS pathogenesis [43]. It is worth noting that in our patients with long QT, we did not observe likely pathogenic, nor pathogenic variants of the gene panels for congenital LQTS.

### 4.3. Truncating Variants

The p.Arg81AlafsTer15 variant was detected in patient 5, showing initial signs of DCM at 18 years. Familiar segregation analyses also confirmed the presence of the variant in her mother and maternal aunts, both of them presenting with DCM. This variant was previously reported by Ortiz-Genga et al. as being associated with the same cardiomyopathic phenotype [23]. 

The p.Leu2051ThrfsTer25 variant was identified in patient 13 and has neither been reported previously in the literature nor listed in genomics databases. According to the ACMG guidelines, it is classified as pathogenetic on the basis of a loss-of-function mechanism. The patient showed congenital UAV and the dilatation of the ascending aorta. The same variant was also detected in his mother who refused cardiac screening. The maternal aunt presented DCM at her 4th decade of life and was not available for molecular testing.

## 5. Conclusions

*FLNC* variants in the pediatric population show wide phenotypic variability. We demonstrated a potential genotype–phenotype correlation of the p.Ala1186Val variant in causing severe and early-onset RCM associated with musculoskeletal involvement. Moreover, we identified a relatively frequent finding of LQTS in three out of fourteen patients (21.4%) and one patient with a QTc value within the upper normal limit. The latter might represent a specific red flag for diagnosing this very specific category related to *FLNC* variants. The major limitations of this study include the relatively small sample size, potential high genetic heterogeneity, incomplete penetrance, age-related penetrance, and variable expressivity of the *FLNC* gene. Accurate and regular cardiac screenings of patients and asymptomatic relatives are highly recommended.

## Figures and Tables

**Figure 2:**
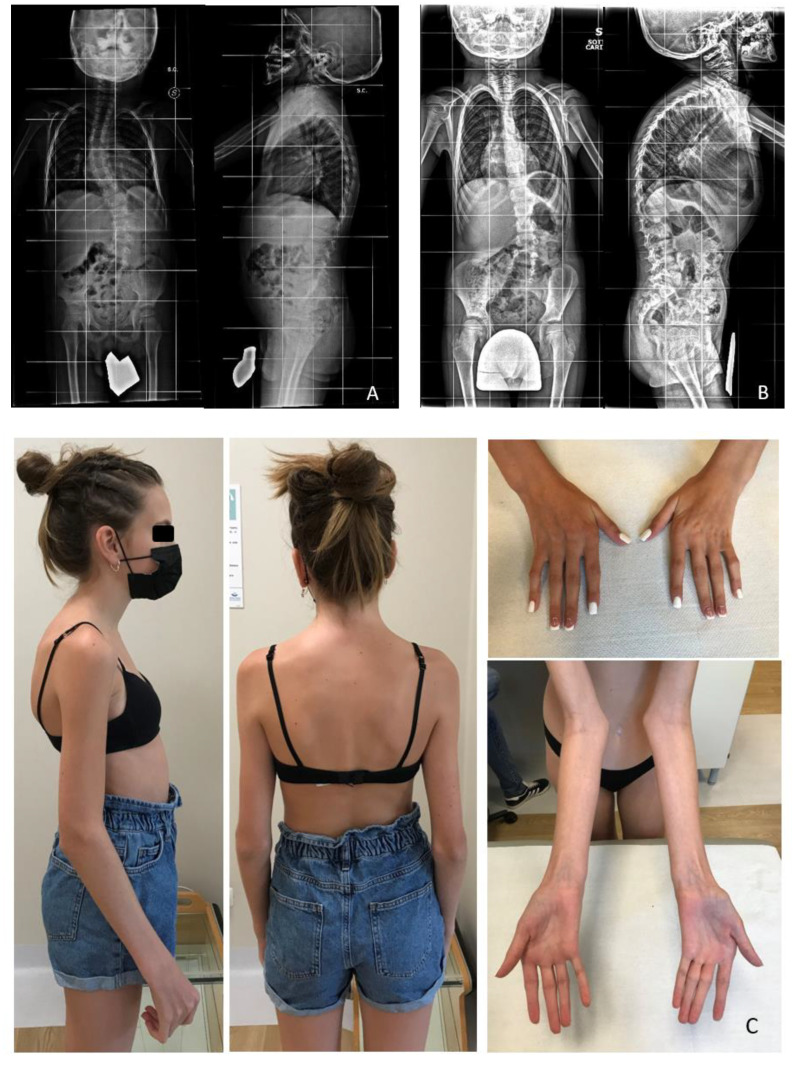
(**A**,**B**) include anteroposterior and lateral vertebral and pelvic X-Rays of patients 7 and 8 respectively at the age of 4 years old. It shows bilateral coxa valga, lumbar scoliosis, and thoracic kyphosis. (**C**) includes musculoskeletal findings of patient 14 with arachnodactyly, camptodactyly of fingers, simplified dermatoglyphic changes, ulnar deviation of the hands, restricted movements, pterygium like changes in elbows and neck, mild scoliosis, and moderate thoracic kyphosis.

**Table 1:** A summary of phenotypic and genotypic characteristics in the studied cohort of pediatric patients with *FLNC* variants. A detailed clinical report is included in Supplementary Material S1.

	Summary of Phenotypic and Genotypic Characteristics in the Studied Cohort of Patients with *FLNC* Variants																	
**Patient**	**Main Cardiac Phenotype**	**Other**	**Arrhythmic Events** **and Conduction System Disorders**	**Muscular/Multisystemic**	**Outcome**	**Age**	**Sex**	**Age at Dx**	**CPK**		**Chromosomal Microarray Analysis**			**Inh**		**CNV Interpretation**	**Reference**	**Family History**
1	DCM/LVNC	Severe LV dysfunction	-	Palpebral ptosis	HT list, Lost in FUP	7 y	M	2 month	N		arr[hg19] 7q32.1(128,470,838-128,521,431)x1 mat			mat		-	Current study	Mother: EF 48–50%
	**Main Cardiac Phenotype**	**Other**	**Arrhythmic Events**		**Outcome**	**Age**	**Sex**	**Age at Dx**	**CPK**	**c-Notation**	***p*** **-Notation**	**Protein Domain**	**Variant Type**	**Inh**	**ACMG Criteria**	**Variant Interpretation**		**Family History**
2	DCM	ESHF	QTc 450msec	Unexplained severe hypotonia after AG	BH, Infant Jarvik, died	4 y	F	2 y and 9 month	high	c.4069G>A	p.Gly1357Arg	ROD1	Missense	Pat	PM2, PP3, BP1	VUS	Current study	Father: EF 50%, IVS 12 mm, no LGE in CMR. Pat GF: G?, Ph: cardiac?, progressive myopathy
3	DCM/EFE	ESHF	-	-	HT	9 y	F	9 y	N	c.4076G>T	p.Gly1359Val	ROD1	Missense	Pat	PM2, PP3, BP1	VUS	Current study	Father: G+, Ph?
4	LV hypertrabeculation	-	Mild LQT	Mild scoliosis, distal BHSJ	Alive and well	15 y	M	15 y	N	c.6751A>C	p.Thr2251Pro	ROD2	Missense	De novo	PM2, PP3, BP1	VUS	Current study	-
5	DCM	EF 52% and diffuse LGE in CMR	-	-	Alive and well	21 y	M	18 y	N	c.241delC	p.Arg81AlaTer15	ABD	Frameshift	Mat	PVS1, PM2, PP3	LP	(Ortiz-Genga et al., 2016) [23]	Mother, mat aunts: G+, Ph+: DCM, ICD. No SCD. Mat GM: G+, Ph- (80 y old)
6	Familial DCM	Normal imaging	AVB-II	Duane ocular defect, mild scoliosis, flat feet, BHSJ	Alive and well	17 y	M	16 y	N	c.6662T>C	p.Val2221Ala	ROD2	Missense	Mat	PM1, PM2, PP3, BP1	VUS	Current study	Mat: DCM. Mat GF: DCM. Mat uncle: DCM, diffuse LGE
7	RCM	Chronic HF	AVB-I, LQTS	Arthrogryposis, LGD (Figure 2A)	HT waiting list	9 y	M	3 y and 6 month	Increased	c.3557C>T	p.Ala1186Val	ROD1	Missense	De novo	PP5, PM2, PP3, BP1	LP	(Kiselev et al., 2018) [22]	Negative
8	RCM	Chronic HF	-	LGD (Figure 2B)	HT waiting list	2 y	M	1 y	Increased	c.3557C>T	p.Ala1186Val	ROD1	Missense	Pat	PP5, PM2, PP3, BP1	LP	(Kiselev et al., 2018) [22]	Father: RCM dx at 18 y, musculoskeletal abnormalities
9	RCM	ESHF, pulmonary hypertension	LQTS	Arthrogryposis, LGD	VAD, HT	13 y	F	11 y	N	c.7570T>C	p.Ser2524Pro	ROD2	Missense	Not Mat	PM2, PP3, BP1	VUS	Current study	
10	HCM	-	-	-	Alive and well	13 y	F	11 y	N	c.3799C>G	p.Arg1267Gly	ROD1	Missense	Pat	PM2, PP3, BP1	VUS	Current study	Sister: SCD at 7 y old. Father: syncope, normal cardiac screening. No recent data.
11	HCM	-	-	-	Alive and well	14 y	M	13 y	N	c.1102G>A	p.Val368Met	ROD1	Missense	Pat	PP3, BP1	VUS	Current study	Father: HCM, IVS 14 mm
12	Congenital AVB, ASD	ASD	Congenital AVB III	Microsomy	Alive	4 y	F	Prenatal onset	N	c.7450G>A	p.Gly2484Ser	ROD2	Missense	Mat	PP3, BP1	VUS	(Verdonschot et al., 2020) [8]	Mother: G+, Ph?
13	Congenital UAV	Ascending aortic dilatation	-	Distal BHSJ	Alive and well	11 y	M	10 y	N	c.6151_ 6161del	p.Leu2051ThrfsTer25	ROD2	Frameshift	Mat	PVS1, PM2, PP3	Path	Current study	Mother: G+, Ph? Refused echo. Maternal aunt: G?, Ph: CMP
14	RCM	-	AVB-I, QTc at upper limits	Arthrogryposis, LGD restricted jaw movements (Figure 2C)	HT waiting list	14 y	F	7 y	N	c.4628G>C	p.Arg1543Pro	ROD2	Missense	De novo	PS2, PM2, PP3	LP	Currentstudy	

Abbreviations: AG, acute gastroenteritis; ASD, atrial septal defect; AVB, atrioventricular block; BH, Berlin heart; BHSJ, benign hypermobility spectrum of the joint; CMP, cardiomyopathy; CMR, cardiac magnetic resonance; CNV, copy number variant; DCM, dilated cardiomyopathy; Dx, diagnosis; EF, ejection fraction; EFE, endomyocardial fibroelastosis; ESHF, end-stage heart failure; F, female; FUP, follow-up; G, genotype; GF, grandfather; GM, grandmother; HCM, hypertrophic cardiomyopathy; HF, heart failure; HT, heart transplantation; Inh, inheritance; IVS, interventricular septum; LGD, limb-girdle dystrophy; LGE, late gadolinium enhancement; LP, likely pathogenic; LQT, long QT; LQTS, long QT syndrome; LV, left ventricular; LVNC, left ventricular non-compaction; mat, maternal; M, male; N, normal; pat, paternal; Ph, phenotype; RCM, restrictive cardiomyopathy; SCD, sudden cardiac death; UAV, unicuspid aortic valve; VUS, variant of uncertain significance; y, years.

## Data Availability

The data presented in this study are available on request from the corresponding author. The data are not publicly available due to institutional research policies.

## Supplementary Materials

The following supporting information can be downloaded at: https://www.mdpi.com/article/10.3390/jcdd9100332/s1. Supplementary Material Data_S1: Includes detailed clinical description of the study cohort., the list of analyzed genes, the NGS workflow and the sequencing Sanger of singleton samples (S1). Supplementary Figure S1. Next-generation sequencing (NGS) workflow of the study patients. Supplementary Figure S2. Sanger sequencing of singleton samples.
