# Supplementary material for: Cardiovascular Involvement in Pediatric FLNC Variants: A Case Series of Fourteen Patients
Source: J Cardiovasc Dev Dis. 2022 Sep 30;9(10):332. doi: 10.3390/jcdd9100332 (PMC9604120; doi:10.3390/jcdd9100332)
Supplement: Supplementary file 1 [file jcdd-09-00332-s001.zip › jcdd-1926128-supplementary.pdf]

Journal of
Cardiovascular Development and Disease

MDPI

Cardiovascular Involvement in Pediatric *FLNC* Variants: A Case Series of Fourteen Patients

1. Detailed clinical features of the reported cohort

1.1 Patient 1: Male. The patient was born of non-consanguineous parents (Gipsies) at the 34th week of gestation by a natural vaginal delivery. His birth weight was 1860 g (5th-10th percentile), with a length of 44 cm (10th-25th percentile), and his occipitofrontal circumference (OFC) was 31 cm (10th-25th percentile). In his second month of life, he presented with a severe form of dilated cardiomyopathy (DCM). Echocardiography showed left ventricular non-compaction (LVNC). He was admitted due to reluctance to feed and failure to thrive. His metabolic investigations and CPK levels were normal. The left ventricle was severely dilated and dysfunctional (ejection fraction—EF: 20-25%), with left atrial enlargement (35 ml/m2) and severe mitral regurgitation (MR). The child had several admissions for recurrent acute heart failure (HF). He was on a heart transplantation (HT) waiting list with maximal anti-HF treatment. His last clinical evaluation, at an age of 5 years old, showed relatively stable DCM (EF: 35%) and persistent severe MR. General physical and neurological examinations excluded signs of skeletal myopathy, except for monolateral palpebral ptosis, which was also present in the mother. He normally reached developmental milestones. His growth parameters at his last cardiogenetic evaluation (5 years old) were as follows: 15.8 kg (10th-25th percentile) and 105 cm (10th-25th percentile). Maternal echocardiography showed mild ventricular dysfunction (EF: 48-50%). A next-generation sequencing (NGS) custom panel for cardiomyopathies (CMPs) was negative for pathogenic variants. A CMA (chromosomal microarray analysis) test, with a single-nucleotide polymorphism (SNP) array technique, showed a maternally inherited microdeletion in the 7q32.1 chromosomal region, extended by about 50.6 Kb (128,470,838-128,521,431). The deletion involved *FLNC*. The patient was lost in the follow-up.

1.2 Patient 2: Female. The patient was born of non-consanguineous parents. Her psychomotor development was normal. She was admitted at the age of 3 years old, after presenting a symptom of acute gastroenteritis, where she was diagnosed with end-stage HF secondary to DCM that needed a mechanical assistance device. Echocardiography showed very severe left ventricular (LV) dysfunction with an EF < 15%, hypertrabeculation, and smoky cavity. The clinical course was characterized by prolonged cardiocirculatory assistance with the consecutive use of Levitronix, a Berlin heart, and an infant Jarvik. She was on a list for an HT. She showed cerebral hemorrhage with hypertensive hydrocephalus, H1N1 bocavirus infection, and macrophage activation syndrome. The patient had a slow progressive improvement in LV function, and therefore the Jarvik system was explanted. Her clinical condition was later complicated by a fungal infection due to candida meningoencephalitis. Her cardiovascular status worsened with rapidly progressive myocardial dysfunction and then cardiac arrest. Her CPK levels were elevated. A custom NGS panel for CMPs showed a paternally inherited variant of *FLNC:* c.4069G>A, p.Gly1357Arg. Her father presented with mild LV dysfunction, increased thickness of the interventricular septum (IVS), and no late gadolinium enhancement (LGE) in cardiac magnetic resonance (CMR). Her paternal grandfather, who refused a physical examination and genetic testing, was said to be affected by progressive myopathy.

1.3 Patient 3: Female. The patient was born of non-consanguineous parents at the 41st week of gestation by a normal vaginal delivery. Her birth weight was 3600 g (50th-75th percentile), with a length of 52 cm (50th percentile) and an OFC of 36 cm (50th-75th

percentile). DCM with severe LV dysfunction was diagnosed at 3 months of life. Myocardial biopsy ruled out inflammatory myocarditis. A Berlin heart mechanical assistance device was initially implanted, and she then had an HT at the age of 8 months. A histological examination of the heart showed stretching of the myocardial cells, mild ventricular septal interstitial fibro-edematous changes, and endomyocardial fibroelastosis (EFE) of the left ventricle. Since her HT, she has been periodically monitored and always been in fair condition. Her CPK levels have always been normal. Her growth parameters at her last cardiogenetic evaluation (9 years old) were as follows: 31 kg (50th-75th percentile) and 145 (97th percentile). A custom NGS panel for CMPs showed a paternally inherited variant of *FLNC:* c.4076G>T, p.Gly1359Val. Her father refused to undergo a scheduled echocardiographic test. Her paternal aunt had DCM at the age of 47 years old.

1.4 Patient 4: Male. His family history was negative for cardiovascular diseases. The patient was born of non-consanguineous parents at the 39th week of gestation by a normal vaginal delivery. His birth weight was 3600 g (50th-75th percentile), with a length of 52 cm (50th percentile) and an OFC of 36 cm (50th-75th percentile). He has always been in good health and regularly participated in competitive sport. At 13 years of age, ECG screening showed prolonged QTc and high voltage measures. CMR at 14 years of age (weight of 57 kg and a height of 177 cm) showed normal size and function of the left ventricle with medial and apical hypertrabeculation, abnormality in papillary muscles insertion, and multiple myocardial crypts in the junction of the lower medio-basal septal wall. A Holter ECG showed a QTc of 480-500 msec at higher heart rates. His CPK levels were normal. The clinical evaluation showed small joints hypermobility and mild scoliosis. His growth parameters at the last cardiogenetic evaluation (15 years old) were as follows: 65.50 kg (50th-75th percentile) and 178 cm (75th-90th percentile). A custom NGS panel for CMPs showed a de novo variant of *FLNC:* c.6751A>C, p.Thr2251Pro.

1.5 Patient 5: Male. The patient was part of a large family with DCM. He showed normal growth and development. His CPK levels were not measured. At 18 years old, CMR showed an EF of 52% and diffuse LGE distribution. A custom NGS panel for CMPs showed a maternally inherited variant of *FLNC*: c.241delC (p.Arg81Alafs*15).

1.6 Patient 6: Male. His family history was positive for DCM on the maternal side. His maternal grandfather was diagnosed with DCM (EF: 20-25%) at 56 years old. A Holter ECG showed first-degree atrioventricular block (AVB). He had an ICD implantation at 68 years of age. A custom NGS panel for CMPs showed two variants: *FLNC* c.6662T>C p.(Val2221Ala) and *TTN* c.13726G>T, p.(Glu4576Ter). His maternal uncle had acute heart failure (EF: 19%) at 49 years of age. CMR showed apical midventricular hypertracebeculation, and LGE confirmed stria in the mid-basal segments of the left ventricle. His maternal uncle's genotype was positive for *FLNC* variant c.6662T>C, p.Val2221Ala. His mother had been in regular cardiac screening since the age of 40. CMR at 49 years showed diffuse hypertrabeculation, slightly dilated left ventricle, and an EF of 50%. She had a loop recorder that registered sinus tachycardia, monomorphic premature ventricular contractions (PVCs), and rare premature supraventricular contractions. The last Holter ECG at age 50 showed first-degree AVB, rare premature supraventricular contractions, and numerous monomorphic PVCs. She is on medications of beta blocker (bisoprolol) and enalapril. The maternal genotype confirmed both familial variants: *FLNC* c.6662T>C, p.(Val2221Ala) and *TTN* c.13726G>T, p.(Glu4576Ter). The patient was born at the 39th week of gestation by a C-section due to breech presentation. His birth weight was 3150 g (10th-25th percentile), with a length of 49 cm (10th-25th percentile) and an OFC of 34 cm (10th-25th percentile). He has always been in good health. His first cardiac checks were at 16 years old as part of a screening

program for relatives of those with CMPs. Echocardiography was normal; a Holter ECG showed that he had second-degree AVB. The clinical evaluation showed a high-arched palate, distal joints hypermobility, mild scoliosis, and bilateral flat foot. His CPK levels were normal. His growth parameters at the last cardiogenetic evaluation (17 years old) were as follows: 68 kg (25th-50th percentile) and 174 cm (25th-50th percentile). Segregation analysis confirmed both familial variants: *FLNC* c.6662T>C, p.(Val2221Ala) and *TTN* c.13726G>T, p.(Glu4576Ter). At the last evaluation the young proband presented only the arrhythmic spectrum without myocardial involvement yet, similar to his older relatives who were affected by CMPs after their fourth decade of life.

1.7 Patient 7: Male. His family history was negative for cardiovascular diseases. The patient was born of non-consanguineous parents at the 38th week of gestation by a normal vaginal delivery. His birth weight was 3350 g (25th-50th percentile). Hypertonia and arthrogryposis were reported at birth. On the occasion of an ECG at the age of 3 years old, severe long QT syndrome (LQTS) (495 msec) was observed; echocardiographic screening showed restrictive cardiomyopathy (RCM) with severe biatrial dilatation and increased brain natriuretic peptide (BNP). Since then, the patient has been under constant cardiac monitoring, and he is on an HT list. He is on medication of nadolol, enalapril, and furosemide. His last echocardiography (at 12 years old) showed normal left ventricular function, significant left atrial dilation (vol. of 50 ml/m2), minimal MR, slightly reduced right ventricle size, and minimal tricuspid valve insufficiency. A Holter ECG detected first-degree AVB and prolonged QT (461-472 msec). The condition was not isolated, but instead showed multisystemic involvement: limb-girdle muscle weakness, severe kyphoscoliosis (Figure 2A), short neck, semi-flexion of elbows, facial weakness, and progressive convergent strabismus. Inability to obtain a sitting position independently. He had surgery for kyphoscioliosis at 11 years of age. His CPK levels were always increased. Spirometry revealed severe restrictive respiratory failure. A custom NGS panel for CMPs showed a de novo variant of *FLNC:* c.3557C>T, (p.Ala1186Val).

1.8 Patient 8: Male. His father was diagnosed with RCM at 18 years of age. CMR at 40 years of age showed severe biatrial dilatation (right > left), pericardial effusion (10-11 mm), LGE in left ventricular myocardium (non-ischemic pattern), and biatrial as well as atrioventricular valve enhancement. He was on furosemide, digoxin, warfarin, and potassium canrenoate treatment. His father's physical examination showed limb-girdle muscle weakness, dystrophic aspect, reduced muscular mass, right-sided deviation of the mouth, joint stiffness in major joints (especially the shoulders and elbows), and mild scoliosis. The patient was born of non-consanguineous parents at the 39th week of gestation by a C-section. His birth weight was 3250 g (10th-25th percentile), with a length of 44 cm (5th-10th percentile) and an OFC of 35 cm (25th-50th percentile). He had developmental delays since the first months of his life. At one year of age he was diagnosed with RCM with moderate biatrial dilatation. Cardiac catheterization showed elevated end-diastolic pressure. Dysmorphic features included frontal bossing, sunken eyes with big cornea, epicanthal folds, convergent squint, depressed nasal bridge, short nose, microstomia and mild micrognathia, severe high-arched palate, short neck, *pterygium colli*, limb-girdle muscle weakness, narrow chest, prominent sternal bone, mild brachydactyly, and adducted thumbs. A neuromuscular evaluation showed restricted movements of the shoulder with limited excursion in the elevation of the upper limbs, pelvic girdles with muscle rigidity, hypertonia of all limbs, pes cavus, adducted left forefoot, and slight left thoracic kyphosis (Figure 2B). At his last echocardiographic evaluation he presented with slightly dilated hepatic veins and inferior cava vein, biatrial dilatation, greater on the left, and a restrictive transmitral flow pattern. His CPK levels were always slightly increased. He was on furosemide

and enalapril treatment. His growth parameters at the last cardiogenetic evaluation (3 years old) were as follows: 13.5 kg (10th-25th percentile) and 94.2 cm (3rd-10^{th}percentile). A custom NGS panel for CMPs showed a paternally inherited variant of *FLNC:* c.3557C>T, p.(Ala1186Val). As mentioned above, his father was phenotypically positive for both musculoskeletal and RCM features.

1.9 Patient 9: Female. The patient was born at the 39th week of gestation by a C-section due to breech presentation. Her birth weight was 4100 g (90th-97th percentile). At birth she had hypertonia and arthrogyposis, specifically flexion contractures of the hands and lower limbs ("*clenching hands*"). Her psychomotor development was normal. At 11 years of age, she presented with a severe form of RCM onset with ascites and dyspnea. Her initial symptoms were easy fatigue and chest pain. Echocardiography showed severe biatrial dilatation, moderate tricuspid regurgitation, pulmonary hypertension, and inferior vena cava dilatation. At a Holter ECG she also had a prolonged QTc (521 msec). She started treatment with beta blockers. Due to the severe cardiac phenotype, the patient received an HT at 13 years of age. A multisystemic physical examination revealed limb-girdle muscle weakness, hypotrophy and mild weakness of pectoral muscles, stiff spine, rotoscoliosis and semi-fixed neck deviation to the left, left eye adduction deficit in the oculomotor nerve. Muscle magnetic resonance imaging (MRI) of her lower and upper limbs and her CPK levels were normal. At clinical evaluation she presented with a triangular face, strabismus, prominent upper central incisors, arched upper lip, triangular chin, arched palate, crumpled ear helix, stiff neck, long fingers, and stiffness at elbow extension. A postoperative brain MRI was suggestive for bilateral occipito-parietal posterior reversible encephalopathy syndrome **(**PRES). Her growth parameters at the last evaluation (13 years old) were as follows: 40 kg (10th-25th percentile) and 152 cm (10th-25th percentile). A custom NGS panel for CMPs showed an *FLNC* variant of c.7570T>C, (p.Ser2524Pro). This variant was not maternally inherited. The unaffected father was not available for a genetic analysis.

1.10 Patient 10: Female. Her sister died suddenly at 7 years old. The autopsy described left ventricular myocardial hypertrophy, inflammatory infiltrate, and partial myocardial replacement with fatty tissue. Her father had repeated syncope but normal echocardiography. The father's CMR showed atria at the upper limits and right ventricular (RV) bulging of the free wall in correspondence with trabecular insertions. The patient was born of non-consanguineous parents at the 38th week of gestation by a normal vaginal delivery. Her birth weight was 3300 g (50th-75th percentile), with a length 50 cm (50th percentile). At 11 years old echocardiography showed an initial hypertrophic cardiomyopathy (HCM) with slightly increased myocardial thickness of the lateral and mid-apical wall of the left ventricle. Normal CMR. Her CPK levels were always normal. Her growth parameters at the last cardiogenetic evaluation (14 years old) were as follows: 72 kg (75th-97th percentile) and 159 cm (25th–50th percentile). A custom NGS panel for CMPs showed a paternally inherited variant of *FLNC:* c.3799C>G, p.(Arg1267Gly). The variant was confirmed in the deceased sister in a post mortem analysis on autoptic tissues.

1.11 Patient 11: Male. His father had concentric HCM and normal systolic function that was diagnosed at 40 years of age. The patient was born of non-consanguineous parents at the 37th week of gestation by a normal vaginal delivery. His birth weight was 2250 g (third percentile). He had always been in good health and regularly participated in sport. At 12 years of age, he was diagnosed with mild ventricular hypertrophy as part of a cardiac screening program for relatives of those with CMPs. CMR at 13 years of age (weight of 64.70 kg and a height of 164.5 cm) showed left ventricular normal size and mid-baseline hypertrophy of IVS 10-11 mm (Z-score: + 2). Global

systolic function was preserved (EF: 70%). His CPK levels were always normal. He was being treated with beta blockers. His growth parameters at the last cardiogenetic evaluation (14 years old) were as follows: 66 kg (75th-97th percentile) and 173.5 cm (50th-75th percentile). A custom NGS panel for CMPs showed a paternally inherited variant of *FLNC:* c.1102G>A, p.Val368Met.

1.12 Patient 12: Female. Family history was negative for cardiovascular diseases. The patient was born of non-consanguineous parents at the 29th week of gestation by a C-section due to severe pericardial effusion and complete AVB. Her birth weight was 1000 g (25th percentile), with a length of 31 cm (< third percentile) and an OFC of 26.5 cm (10th-25^{th}percentile). At birth she presented with atrial septal defect (ASD), complete AVB, and respiratory distress syndrome. She underwent ASD repair and pacemaker (PMK) implantation at 1 year and 5 months old. At her last echocardiographic evaluation (6 years old), she presented with residual slight regurgitation of the atrioventricular valves with a mild increase in right ventricular systolic pressure (RVSP). Her CPK levels were always normal. Her growth parameters at the last cardiogenetic evaluation (6 years old) were as follows: 17.75 kg (3rd-25th percentile) and 104.5 cm (< third percentile). A custom NGS panel for CMPs showed a maternally inherited variant of *FLNC:* c.7450G>A p.(Gly2484Ser). The mother refused to perform a scheduled echocardiographic check-up.

1.13 Patient 13: Male. His family history was positive for CMPs, e.g., the maternal aunt. The patient was born of non-consanguineous parents at the 39th week of gestation by a C-section. His birth weight was 3520 g (50th percentile), with a length of 49.2 cm (10th-25th percentile) and an OFC of 34.8 cm (10th-25th percentile). At two years old, an echocardiographic screening showed unicuspid aortic valve (UAV) with mild steno-regurgitation and moderate to severe ascending aorta dilatation. The diameter of the aorta increased from 28 mm to 37 mm in 6 years (max Z-score: 7.5 at the last echocardiography at 11 years of age). The clinical evaluation showed dystrophic skin scars on the face, trunk, and limbs, auricles with soft and deformable cartilage, and a Beighton score of 5/9. He also has moderate myopia (-5 diopters bilateral). His CPK levels were normal. His growth parameters at the last cardiogenetic evaluation (11 years old) were as follows: 32.5 kg (3rd-10th percentile) and 141.5 cm (3rd-10th percentile). He was on medication of angiotensin II receptor blockers since the age of 6 years old. A custom NGS panel for CMPs showed a maternally inherited variant of *FLNC:* c.6151_6161del p.(Leu2051ThrfsTer25). An NGS test excluded variants in genes involved in major forms of connective tissue diseases.

1.14 Patient 14: Female. Her family history was negative for cardiovascular diseases. The patient was born of non-consanguineous parents at the 39th week of gestation by a normal vaginal delivery. Her birth weight was 3300 g (55th percentile), with a length of 54 cm (> 99th percentile). At 8 years of age, an echocardiogram was performed during a medical visit for sport screening, and a suspicion of RCM was raised. CMR showed biatrial dilation, normal size and function of both ventricles, diastolic disfunction, and negative LGE. The diagnosis was confirmed by cardiac catheterization, which showed elevated end-diastolic pressure with normal pulmonary vascular resistance. The patient was put onto a heart transplantation (HT) waiting list. Her CPK levels were always normal.

At her last evaluation (14 years old), she was still on an HT waiting list, as well being on beta blockers, ACE inhibitors, and diuretics. BNP of 572 pg/ml (n.v. < 217 pg/ml). Her growth parameters were as follows: 42.5 kg (5th percentile) and 162.5 cm (66th percentile). At 24 hours of ECG Holter monitoring she always showed first-degree AV block at lower rates and a QTc of upper normal values (450-460 msec).

She had normal cognitive and language development, a nasal voice, a dysmorphic palate with median raphe, a prominent and tubular nose, frontal bossing, deep sunken eyes, epicanthus, mild bilateral ptosis, restricted jaw movements, muscular dystrophy, kyphoscoliosis, mild distal arthrogryposis with restricted interphalangeal movements, camptodactyly of intermediate fingers, and excessive finger ulnar deviation (Figure 2C).

A custom NGS panel for CMPs showed a de novo variant of *FLNC:* c.4628G>C, p.Arg1543Pro, and a negative SNP array.

Supplementary Material Data_S1

CMP gene list

ABCC9, ANK2, ALPK3, *ANKRD1, ACTC1, ACTN2, BAG3, CACNA1C, CALM1, CALM2, CALM3, CALR3, CAV3, CTNNA3, CSRP3, DES, DNAJC19, DOLK, DSC2, DSG2, DSP, DTNA, EMD, EYA4, FAH, FHL2, FHL1, FKRP, FKTN, FLNC, FOXRED1, FXN, GATA4, GATA5, GATA6, GATAD1, GFM1, GLA, GLB1, GNPTAB, GUSB, GYG1, HCN4, HFE, HRAS, ILK, JARID2, JPH2, JUP, KCNE1, KCNE2, KCNH2, KCNJ2, KCNJ5, KCNJ8, KCNQ1, KLHL24, KRAS, LAMA2, LAMA4, LAMP2, LDB3, LIAS, LMNA, LZTR1, MAP2K1, MAP2K2, MLYCD, MRPL3, MRPL44, MRPS22, MTO1, MYBPC3, MYOM1, MYOT, MYOZ2, MYPN, MYH6, MYH7, MYL2, MYL3, NEBL, NEXN, NF1, NONO, NRAS, OBSCN, PDLIM3, PKP2, PLD1 ,PLN, PMM2, PPA2, PPCS, PRDM16, PRKAG2, PTPN11, QRSL1, RAF1, RBM20, RIT1, SCN4B, SCN5A, SCO2, SDHA, SGCD, SGCG, SHOC2, SLC22A5, SLC25A3, SNTA1, SOS1, SPEG, SURF1, TAFAZZIN, TBX20, TCAP, TGFB3, TMEM43, TMEM70, TNNC1, TNNI3, TNNT2, TPM1, TRDN, TRIM63, TTN, TTR*, and *VCL.*

Supplementary Figure S1. Next-generation sequencing (NGS) workflow of the study patients. The workflow starts with sequencing, alignment, variant calling, annotation, variant filtering, and variant interpretation as well as classification according to the American College of Medical Genetics and Genomics guidelines.

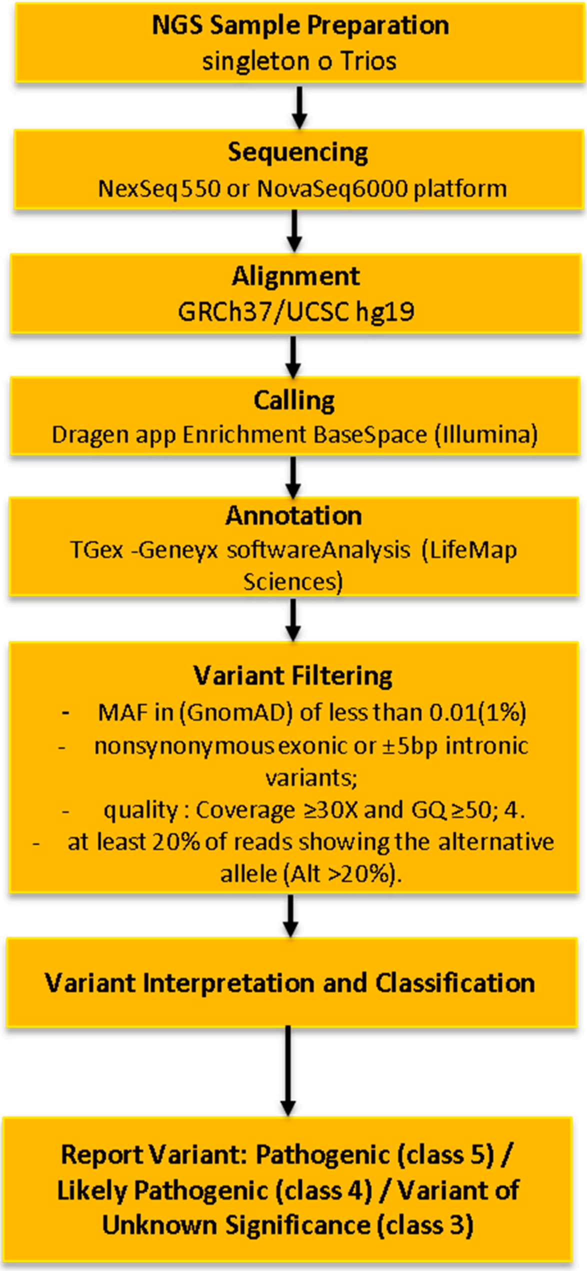

Supplementary Figure S2. Sanger sequencing of singleton samples. The figure panels include Sanger sequencing of patients 2, 3, 4, 8, and 12.

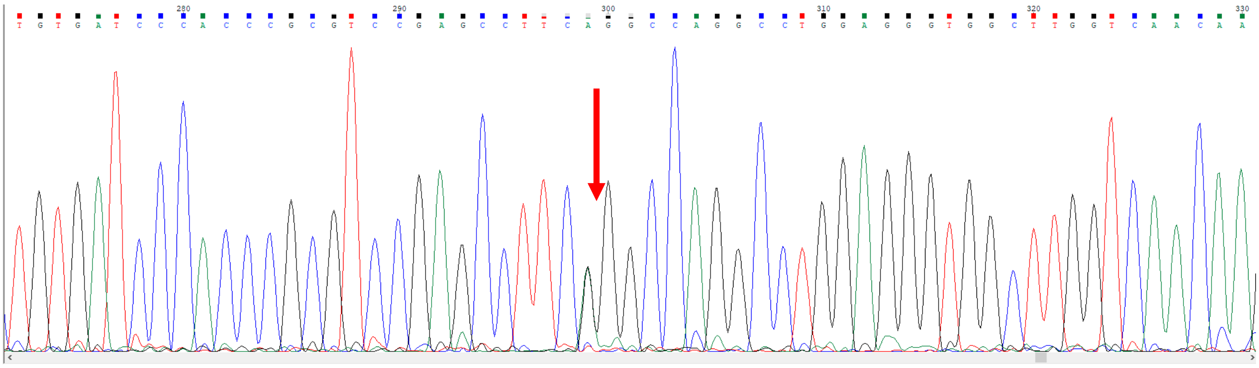

Patient 2: NM_001458.4 (*FLNC*): c.[4069G>A];[=] p.[(Gly1357Arg)];[=].

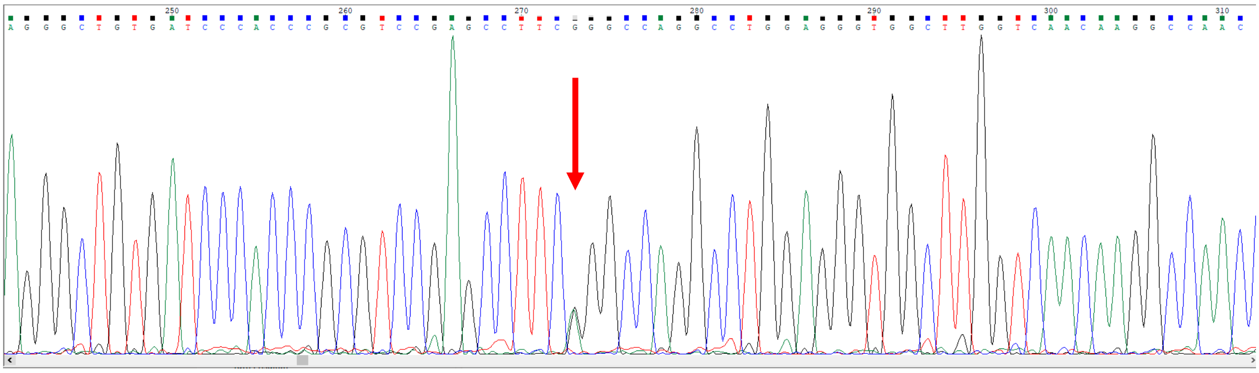

Patient 3: NM_001458.4 (*FLNC*): c.[4069G>A];[=], p.[(Gly1357Arg)];[=].

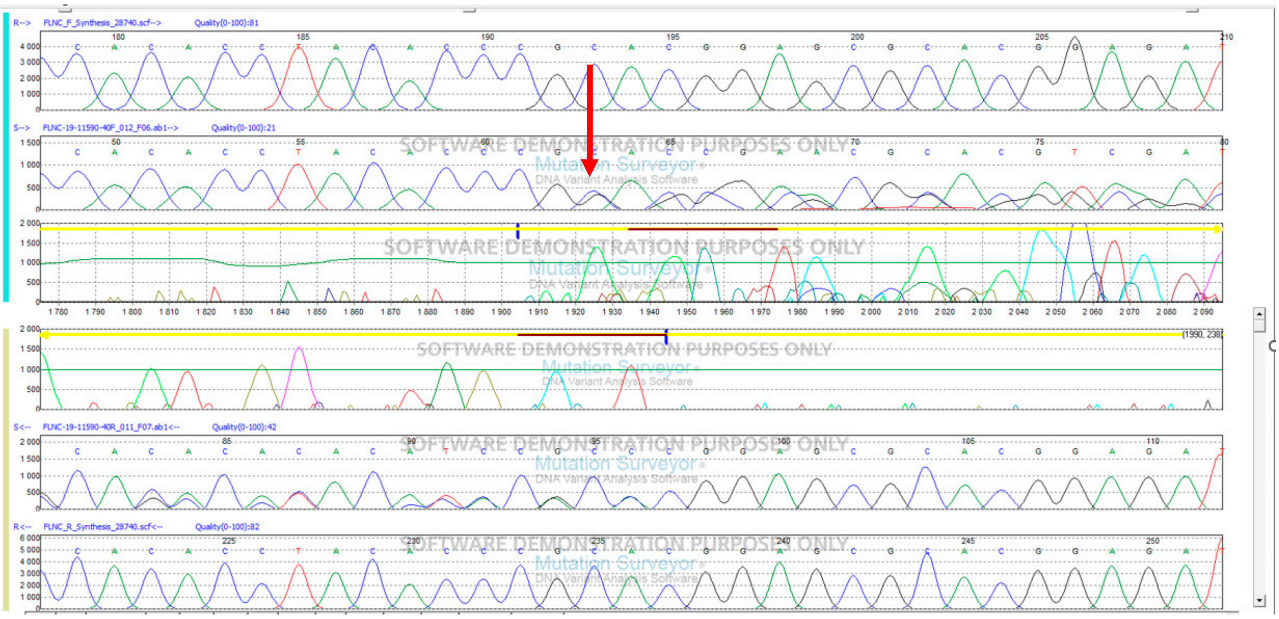

Patient 4: NM_001458.4 (*FLNC*): c.[241delC];[=] p.[(Arg81AlaTer15)];[=].

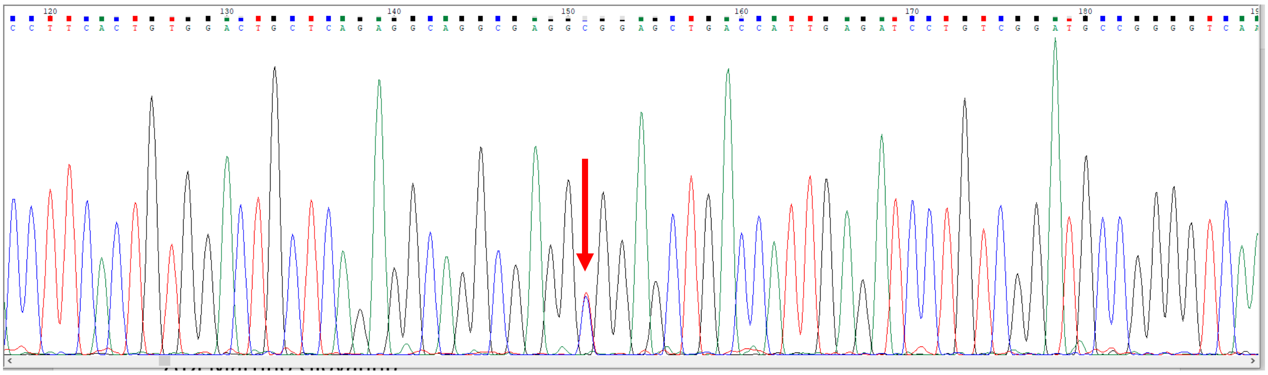

Patient 8: NM_001458.4 (*FLNC*):c.[3557C>T];[=]   p.[(Ala1186Val)];[=].

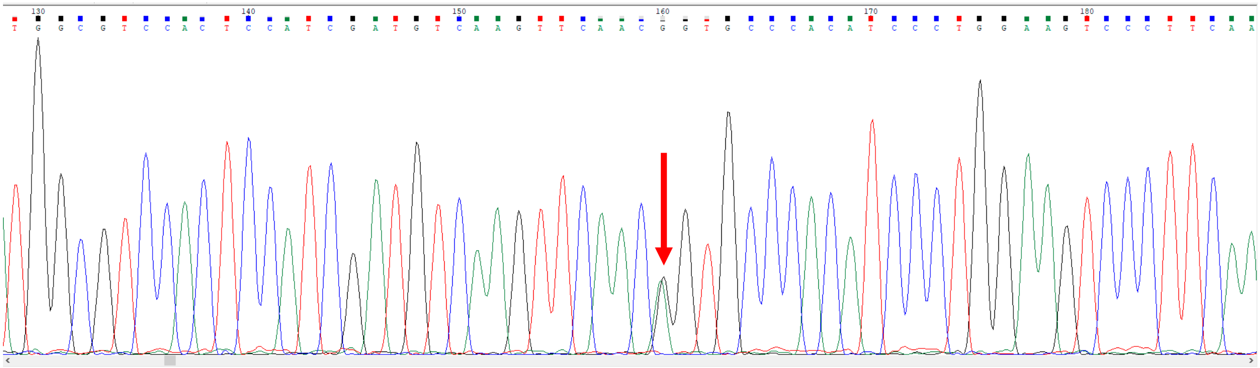

Patient 12: NM_001458.4 (*FLNC*): c.[7450G>A];[=] p.[(Gly2484Ser)];[=].
